# Supplementary material for: Using Diatom and Apicomplexan Models to Study the Heme Pathway of Chromera velia
Source: Int J Mol Sci. 2021 Jun 17;22(12):6495. doi: 10.3390/ijms22126495 (PMC8233740; doi:10.3390/ijms22126495)
Supplement: Supplementary file 1 [file Supplementary Table S1.pdf › ijms-1243821-supplementary/Supplementary Table S1.pdf]

Enzyme	Accession (CryptoDB)	SignalP various versions + TargetP1.1: 3.0	SignalP various versions + TargetP1.1: 4.0 + 4.1	SignalP various versions + TargetP1.1: 5.0	ASAFind (SignalP 3.0)	ASAFind (SignalP 4.1)	ASAFind (SignalP 5.0)	ASAFind + (SignalP 4.1)	ASAFind TargetP 2.0	ASAFind + TargetP 2.0	Protein length (AA)	AA sequence
**ALAS**	Cvel_28814.t1	SP = NO	SP = NO	SP = NO	no SP	no SP	no SP	no SP	no TP	no TP	559	MFSSGTARSFLTFASQEVKKLCPFVGKMGEMSRGKDLSKMAEMCPVASKMDKPIVIVDGDDAKASPAAHGRHYHTSAASSSSEKIGHHDSTPLYGHHHHQGNLVTHGRKSKPQVYLEHFDGSIEKLHKEGNYRIFANLQRHKGNFPNATFRKAETERMVKIWCSNDYLGMGQHPVVLQASHEALEKSGTGAGGTRNISGTMTYHVALEKELADWHGKEAALLFTSGYVANEAAISTIAQLLPNSIIFSDAKNHASMIAGVRNSKCEKKIFRHNDMQHLEELLAAAPADRPKLIVFESVYSMDGAIAPIHAICDLAEKYGAMTYIDEVHAVGMYGPRGAGVCEQIGAADRVDVVNGTLGKAVGVFGGYIAGSAKLVDAVRSYASGFIFTTALPPVVAAGATASIRHLKESSAEREAQHLKVAQLKHMLKSKNLPVMDNPSHIVPIHVGDAALCKAASDELLNAYGMYIQPINYPTVPKGTERLRVTPGPLHSLNDIMTLVDALDDIWTRLNIPRAQEEHAAENPTIDDLFYFAHSNAEIQARARAGVTPEAMQMSMSASA
		TP = NO										
**ALAD1**	Cvel_108.t1	SP = YES	SP = YES	SP = YES	Plastid (hc)	Plastid (hc)	Plastid (hc)	Plastid	Plastid, (hc)	Plastid	402	MRALLLWTSVVGSSGFLQPPVSGPSQRQTPPTELRGLPQSSPFKGVPDSETPGALREELQPLVDLATRPRRNRRNQVFRSLMQETVVRPSNLLQPLFIHADDSAVPIGTMPGMSRLSVKATVDEVAELKSRGVKNVILFPKIDDSLKSNYADECWNPDGLVPTCVKAIKDAHPDVMVYTDIALDPYSSKGHDGVVDEASGRILNDETVAVLCAQALCHAKAGSDMVAPSDMMDGRIGAIRGALDSRGDANAETVGIMAYTAKFASAYYGPFRDALDSHPGFGDKKTYQQDPSNIRESLLETALDNDEGADILMVKPGLPYLDILSRMRDSTELPLAVYHVSGEYAMMKAAAEKGMVNEKNAVMETMKCFKRAGADLIATYYAKDVAKWIQEGAKSGDLEGCF
		cTP = 0,68 YES							SSG-FLQ	SSG-FLQ		
**ALAD2**	Cvel_13826.t1	SP = NO	SP = NO	SP = NO	no SP	no SP	no SP	no SP	no TP	no TP	375	MWSNASSDSSTCGAPVKRTEEGRSVCSTHDDPKRSRGSGDLSVERGPLQIPSRHRLQAGFSGSLLRSWQGVPPLQPSNLVFPVFVVDQEELKEEIRTMPGQFRFGYKTLVRELSSLVEKGLGGVMIFGVVEGGLKDERGTCADSLDGPVIKALQILKRELPQLLLVADVCLCEYTNHGHCGILDQSGRVINKCSVDRIAEIAVAYARAGADVLAPSDMSEGRVMQIKKRLLADSEIAHKAVMSYSAKFASAFYGPFREAAGSAPSFGDRKCYQLPPEARTLGIRASLRDAEEGADFLMVKPASHYLDVVKALHDRVDVPLAVYQVSGEYAMLKFASEAGALDLRTAVLESVASMQRAGASVVITYFAPELLDWLP
		TP = NO										
**ALAD3**	Cvel_36189.t1	SP = NO	SP = NO	SP = NO	no SP	no SP	no SP	no SP	no TP	no TP	523	MVEERFPATRMRRSRMQDFARQLTCEHRLSPADLIQPLFVAEGAQIGPVASMPGIQRHALEQLGAECRRVQALGVPAVALFPVLPPEAKDDSGSAALDPDGLVPRAIRAAKEAAPGLGIIIDIALDPFTDHGHDGVLRADGSVDNDATVELLTKQAILYARAGADVLAPSDMMDGRVGALRTALERDRRFDVLILSYAAKYASAFYGPFRDAVGSRTALIGDKRSYQMDPAGSDEAMREIALDLREGADWVMVKPGMPYLDIVRRAKAEFGVPTDIALSYEAIEVAPEALAGQLAQWHREAWSGLNVTLPHKTAVPAACVDCSERARVAEAVNTLCHLDNGWYGDNTDGEGLLRDLVDNLDVAVANQRILVLGAGGAARGILLPLLQRRPAELVLSNRTPWKPEAIAEAMAEHGRIRPSTHLALKGDRFDLVINAMSVGHSGHFPRLPEGLFDEGAVAYDLSYGAAHAPFAAWARAQGAAVVHDGLGMLVEQAAVSFQLWNAVLPRTDALIATLRAESRSTAV
		mTP = 0,63 YES										
**PBGD**	Cvel_26028.t1	SP = YES	SP = YES	SP = YES	Plastid (hc)	Plastid (hc)	Plastid (hc)	Plastid	Plastid, (hc)	Plastid	375	MVCSGLFVGGLLVSAVSAFTVVDPSQSSLLSRGRRTASRSSLQMALDKPILTIGTRGSMLALAQAHETKRRLGEKFPELAEEGAIELKVISTTGDERLEIALSEIGGKGLFTKELDVALLTKQVDICVHSMKDVPTWLVDGTILPVNLPREDTRDVFISKVAKRIEDLPEGAVVGTASLRRAAQVLYKNPKVKVVNFRGNVQTRLRKLDEKQVDATMLALAGLNRLGMTDVATSVLDHDEILPAVSQGAIGIQCRADDERVQKYIAPLGCAKTKAAVDCERAFLGGLDGNCRTPIAGHAFITEDGRLKFKGMVSSMDGSEFFYVEKEGAVEDGPKMGKEAADEITAQCGEEFLEKLRAATPHNTLPSPHMYEATA
		mTP = 0,638 YES							VSA-FTV	VSA-FTV		
**UROS**	Cvel_15018.t1	SP = NO	SP = NO	SP = NO	no SP	no SP	no SP	no SP	no TP	no TP	434	MNFSCFTFLLNCRPSTPSKELRADRVEEVHFCTPTGGDASPETDLLTHSLYLQSAGVMVSVIPWLAFLCPSSSGHSLLGKRAARRACALMTESSQPSPGEAAPPRGETGTCVVVAGTREESKNGKLKKALSDIIAPEDFEFVEVPCIAAQAGPDRERLVDTLAEEGRRVGAGEKPRFEYAAVTSPEGANVFLECLRSASLTKCPIPVTAVGKSTAEILAKAGVEPVFVPSKATAETLAAELPSVSSSSSTSSSSSSSSSSLSSSSCPGRVLYPTSNLSLGTLERLLKERGFEVTRLETYATVTAQWDEEKTQSAARTQIATFGSPSSVRNWVEKLSLREGGRELLSSQAAACIGETSGRAAEKMEVFREVVWPEKPGMDAWARLTADLVVAHRRRNGSESSPHDASRALLNASPQAADHSGNAASDEELIVGEK
		TP = NO										
**UROD1**	Cvel_14720.t1	SP = YES	SP = YES	SP = YES	Plastid (hc)	Plastid (hc)	Plastid (hc)	Plastid	Plastid, (hc)	Plastid	436	MGVMMRALGSLAVSLFLSSSSAFRLSVSSLPSPVSSQETKRAQGSQPRSCLSMSGTLKNDLMLRAARGETVERTPVWLFRQAGRHLPEYNEYKKQKGKNFLQLLNDPEDVAEVTLQPIRRYDVDAAILFSDILVVLQAFGFDVEMPGGRGITVPSPLADVEDFRRRFPSSDEVDVQMKLGHVLESVSLIKERLEGQVPLIGFSGAPWTLMFYLVGGSSKQNTDRGRQWLERHPEESRKIINGLTDVVIEYLSAQVERGADMLQVFEAMGDFLSPSQFEEFALPSLERIARELKKRHPQVPLLVFPRGACYAIATLQECGYDVVTLDTATDRMSARADLSRAARESGLGKPARVQGNFPVGLLQRRDGVSLEEDIERVRAEAERMLEQ
		cTP = 0,929 YES							SSA-FRL	SSA-FRL		
**UROD2**	Cvel_5098.t1	SP = YES	SP = YES	SP = NO	Plastid (lc)	Plastid (lc)	no SP	Plastid	Plastid, (hc)	Plastid	442	MMMFKSVGAVLVAVSVLEGSGFSVQSRSFLKRPLFGFRSRSSQSSALDMATATLEPIQASDLQGLEFPPQVTEPMSPSEKAELREERRAAMKPDWPLPKNTNVINALKGEEVEAVPVWMMRQAGRYLPEFRAVRAGSEFLKVCQDPGLASEVTVQPRRRYPELDACIIFSDILVVPQAMGQPLQMLPNRGPVFDWRLESPEDIKKLDLTPDVEDSLGYVFDAMYLTKKKVDNEVPIFGFCGAPWTILGYMVEGEGSRTWAETKRWLYDYPEESHKLLEAISRLSAKYLIGQWDSGAQMLQVFDTNAGVLPPHLYEEFGAKYMRMIADLVKAERPQALIVGFPKDMPLASFNDSSYDGIGLSWTADVQEMRERFPNKTLQGNMDAHILYANKDAIRKATHEMVKEFGKKRYVANLGHGMEPRMDPEHAKAFIQSIKEASSELE
		mTP = 0,64 YES		cTP = 0,356 YES					GSG-FSV	GSG-FSV		
**UROD3**	Cvel_31936.t1	SP = YES	SP = YES	SP = YES	Plastid (hc)	Plastid (hc)	Plastid (hc)	Plastid	Plastid, (hc)	Plastid	433	MWKRRLLFLASLSLLLEPAVRVCDAFRFPISMTITTDLPRGNQKTPVVAGKGSAMMARPEREEAQSAEHDLLLRAAKGEQTHRAPVWLMRQAGRYMEEFRKYSDVYPFRQRSETPNIAVELSLQPFRAFGTDGVIMFSDILTPFPAFGIDFDVVKGKGPVFPEPVRTKEDVEMRLRPVEDFGRSLPFVGETLRRLRKETEGRTTLIGFVGSPFTLAAYACEGGGSKECLHMKEMMLRRPETARKFLSFLAEQIGRYVSFQVQNGAQLVQVFESWAHHLGADDWETWAKPYAERVASVAKQDLREAGYDCESVPLIYFANGGSGYLERQRGLKGYNMVCVDWGCGLEEAVDRLGCNQEGGVGISGNVDPAVLRVGSEDDIRQAVRKCIAAGSRSSNGFVLNLGHGVKQGTPEEAVGWMVDEAKRCKFADVSSQS
		TP = NO							CDA-FRF	CDA-FRF		
**CPOX1**	Cvel_21486.t1	SP = YES	SP = YES	SP = YES	Plastid (lc)	Plastid (hc)	Plastid (hc)	Plastid	Plastid, (hc)	Plastid	400	MGRTQLLLFFATVSWASSFQLAGKGGVTGFLGRRKAAQESTRLKGETHEFINPLNPGELTTHPGEEFRKQWEQIILSAQDKICNGISELEGGREFHEDVFQRPNNGGWGRTRVLQNGKVFEKGGVNVSIVKGHLPPGAARQMTSRGHTGLDPSRALEFYACGLSLVIHPHNPMAPTVHCNYRFFQLFDAETKQPLTWWFGGGTDLTPSYLFEEDAEYFHRTLKNVCDRHGEDKYPRFKRWCDEYFLIPHRGETRGVGGIFFDDFNEGGNPEVGKAFASECLDAFNEAYIPLVRKRMDSEFTPEQKEWQQLRRGRYVEFNLLYDRGTKFGLQTPDARYESILMSLPLTSRWEYQAPQGDVEGSWEQRTQQALKNPRDWIPIDDADLKNILERMNSRELQTA
		mTP = 0,737 YES							ASS-FQL	ASS-FQL		
**CPOX2**	Cvel_2641.t1	SP = YES	SP = YES	SP = YES	Plastid (hc)	Plastid (hc)	Plastid (lc)	Plastid	Plastid, (hc)	Plastid	411	MVPLRTLGLALCVLLQELAGGRAFSASTFDVLSSAHGTDRWAKRRGSPRPRGSRRGLGQSLFAAVLPQERGDASVHGVDSTFDIFSSFLRRKQAEILNKLEEVDGKGKFVREPWSQEETGSEGLTAVLEGGDVIEKGACSTSFIKGVLSVDRARAMKQRKGADGWDLKGGETYRAAALSLVFHSRSPLVPTFRADVRYFEVEGKPGWFGGGADLTPYYLFDDDVREFHGFWKEVCNRFSPRSFPVLKRQCDDYFYLPARREHRGVGGIFFDDLVSFESLLGTSETGGDAVEGEQSQSGSREALEFTKAVAEGWLDSWLPIVERRRGLEFTDQEKEWQLIRRGRYLEFNLLYDRGVKFGLAGPAVSTGRPVRVEAVMVSAPPEIKWKYKHEVPEGSEEARLVEVLKKPRDWV
		cTP 0,924 YES							GRA-FSA	GRA-FSA		
**PPOX1**	Cvel_13840.t1	SP = YES	SP = YES	SP = YES	Plastid (hc)	Plastid (hc)	Plastid (hc)	Plastid	Plastid, (hc)	Plastid	566	MRTFTTVLAPAAFCCCAEAFILTSPRHFSRSEQSVSSLQAGPSSASAAGTERTDALVIGAGLSGSSLAFNLMKNGVSDVTVIEANDRVGGNVISMKKDGFQWEEGPNTFQPNNEIVKLAADLGMADELRFADGGLPRWVFWDNVMHKLPGGPQDIPNFNLLSIPGKIRAALGAIGFVGPIPEKEETIREFVCRHLGIEVFEKLIDPFVSGVYAGDPSKLSIEACLPKIKRLESLGGPGLFDGGLLRMQELKKERAQSAADPNMPMVPKAALGSFKNGLRALCEAIAEKLGDRVKLSTRLVNLKPITDPALLAEGYRLRASLLSLNGGGEREVLAKSVAITTPAYRTADLVESMVPAAAALRTIEYPPVASVIMAYPSSSVAASQQPLSGFGHLIPRKNKIRTLGTIWVSSLFPERAPEGWTVLSSYIGGAQDVKIATLNPEQIAAEVDRDMRTTLLKVDAPKPKILGVRLWERAIPQYKLGHLEMLRRLEEDVSKVDGLFLGGNWKTGVAIGNCVAWGGEAASEICAFLDSLSSPSSSGSAAREAAGEMGGEETKRKEEEVVAVGA
		cTP = 0,637 YES							AEA-FIL	AEA-FIL		
**PPOX2**	Cvel_18037.t1	SP = NO	SP = NO	SP = NO	no SP	no SP	no SP	no SP	no TP	no TP	613	MSRAPHVCVIGGGLAGLTTAYTLRRLLPRVRLTILEKADRLGGYIHTHQTRQPVTTAYGNIYGPYAIDLPEPRGRHHVEEGLQFFRMTGRGGSDLLGLIRCLGVEDKVITTAQSRAFFVDYLRLSILWDGMVRKVPSRGDLVRFLPAVLRDLSTKRGEGVDESVESFFRRRTSMSFVDKIIDPICIGSFGVSPSELSARSVFPSLWAAEREGRSLLGGLVRPSSSRKQGGDVEGMYFVPKTDAFHQRVALSGGGIFTFKKGAGFLISSLERGLRAGGRSANESRAPGRSAQEESETVQIRLSTAVAGVRGAKRKKENEKEDQTRAEGQSGAAEVLLESGECLSVDGVVMAVQAPDAVRLLDHPWTRDAWDGRGWESGERTVAVEMGGISHSHTYTVNMGWADSVIPMSLGRTSGVVVPSREWRREGPAQGVLSAHLSSWVFPDYESVFKREARIVVTVRAQSGREWEREKGLFGGQFGCRHEAPEEAAERAQVFAEKVLGIASETEEVWVRQETVPGRLYEDRGKAVLTEGHAEKMKKIHQVRCREIPWMQVVGGDFHGVSASDTVRDARLAAESLARRLRNFPFEGLKEIPEREAEWIQRVPGLTLKDQSEV
		mTP = 0,672 YES										
**FECH1**	Cvel_18167.t1	SP = YES	SP = YES	SP = YES	Plastid (lc)	Plastid (lc)	Plastid (hc)	Plastid	Plastid, (hc)	Plastid	479	MPTKCHHPRLRPAFLPACVLFLGLFPFSPALSFRLHDSKLLDGSRLRPQRRGVRGVRGVEAPELTALSSEVSPSSRCTSSGVRSPPLSEVEGKLGVLFLNLGGPLNLEGVEPFLYNLFADGEIIRLPKGLKWLQPLIAFVISKARAPSSREKYAAIGGGSPILEYTTQQAAAVERLLKSRGIRDVEAFVGMRYAEPFAESVVKEMLQSGVKRAVVVPLYPHYSISTSKSALLSLMSERSFLGLPHTVVPHWYQREGFVEAVQGLIKKEILSIPREAREKEGVHVLFTAHGVPESYIEEGDPYKDQMEHSVALIAEKVQRSVPSSRMPLSHSLSFQSRVGPVTWLQPYTDDEIKKLAEEGVRNLIVVPISFVSEHLETLEEIDQEYREVAEEAGIRHWRRVPALNLDPRFLRDLADLVGDALQSVPVRVEEALDSSCCPALVRSVESALGFEVGGGEGKKGGRVVGVQKEMEEREMSLQR
		mTP = 0,806 YES							ALS-FRL	ALS-FRL		
**FEHC2**	Cvel_26873.t1	SP = YES	SP = YES	SP = YES	Not plastid	Not plastid	Not plastid	Not plastid	Not plastid (SP positive)	Not plastid (SP positive)	459	MSRRVQGRRPRGCTPLLWLGLFVLSLSVRFAVGIGSSSVSVQEPFLKVPPFLSEARFTFKTINARLKEKFHEEMDNIYHRFSSSLKQYGAGRKNRVGVLLVNLGSPDAPTRRGLRKYLREFLSDRRVIEVPRLIWKPILHGIILPFRSPDSAAKYRNVWTEEGSPLISITKRQVARLQDIVDRRYGTVEGGASEGEGEEGNESLPIPMQRSRVLVEWAMRYGTPSVAAGLRKLREEGCRHILVFPLYPQTCAATTTTVFDVVAQELAREKKDRKTGAESWRMFPHLRFVSGYGDHPSYIKALASRVKRHWTKAGKRRRLVMSFHGIPADLIKKGDAYACFCSKTARLLAEELKLGEEEFDMVFQSRFGSTEWLQPYCDERLEEIAAEGHEEVDVICPGFSADCLETLDEIQREYGELFHQAHEKRGTTGRLFYIKALNDSPEGMQMLGDIVDENLQGWL
		TP = NO			SP positive	SP positive	SP positive	SP positive	RFA-VGI	RFA-VGI		

Supplementary table 1: Output from prediction tools used in this work. SP = signal peptide, TP = transit peptide, mTP = mitochondrial transit peptide, cTP = chloroplast transit peptide, (hc) = high confidence, (lc) = low confidence, AA = amino acid
