# Supplementary material for: Using Diatom and Apicomplexan Models to Study the Heme Pathway of Chromera velia
Source: Int J Mol Sci. 2021 Jun 17;22(12):6495. doi: 10.3390/ijms22126495 (PMC8233740; doi:10.3390/ijms22126495)
Supplement: Supplementary file 1 [file ijms-22-06495-s001.zip › ijms-1243821-supplementary/Supplementary Figure S1.pdf]

International Journal of
Molecular Sciences

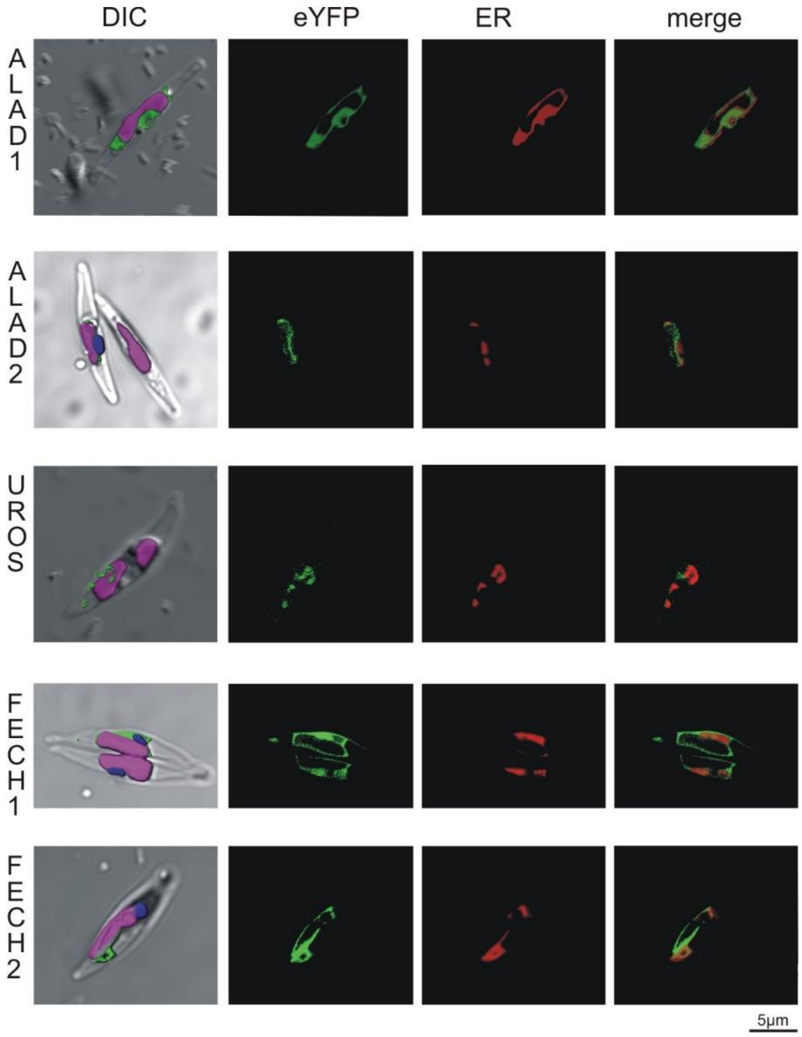

Suplementary figure 1: Heterologous expression of *Phaeodactylum tricornutum* with genes from *Chromera velia* heme pathway enzymes. Even though the majority of *P. tricornutum* cells heterologously expressed with ALAD1, ALAD2, UROS, FECH1 and FECH2 enzymes showed typical “blob-like“ structures patterns, some cells displayed colocalization of eYFP signal (green) with signal of ER-Tracker™ Red (BODIPY® TR Glibenclamide). Plastid autofluorescence (magenta), DAPI (blue).
